# Investigating the effect of polarity of stationary and mobile phases on retention of cannabinoids in normal phase liquid chromatography

**DOI:** 10.1007/s00216-021-03862-y

**Published:** 2022-02-10

**Authors:** Chiara De Luca, Alessandro Buratti, Yannick Krauke, Svea Stephan, Kate Monks, Virginia Brighenti, Federica Pellati, Alberto Cavazzini, Martina Catani, Simona Felletti

**Affiliations:** 1 Department of Chemical, Pharmaceutical and Agricultural Sciences, University of Ferrara, via L. Borsari 46, 44121 Ferrara, Italy; 2 KNAUER Wissenschaftliche Geräte GmbH, Hegauer Weg 38, 14163 Berlin, Germany; 3 Department of Life Sciences, University of Modena and Reggio Emilia, Via G. Campi 103, Modena, 41125 Italy

**Keywords:** Cannabinoids, *Cannabis sativa* L., HPLC, Hemp, Normal phase, Polar-bonded phases

## Abstract

This work reports about a screening of four adsorbents with different polarity employed for the separation of the main phytocannabinoids contained in *Cannabis sativa* L., under normal phase liquid chromatography (NPLC). The effect of polarity and type of interaction mechanisms of the adsorbents (namely Si-, CN-, Diol-, and NH_2_-based SPs) on retention has been investigated under a variety of conditions either by using different combinations of apolar solvents (heptane or hexane) and alcohols (ethanol or isopropanol). The columns have also been employed for the separation of a real cannabis sample.

**Graphical Abstract:**
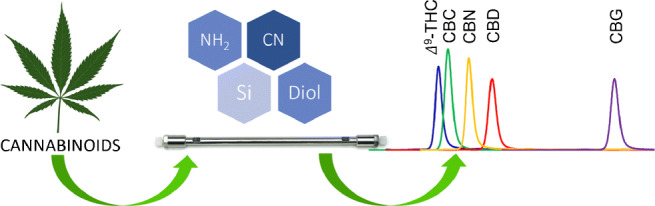
Investigating the effect of polarity of stationary and mobile phases on retention of cannabinoids in normal phase liquid chromatography

Investigating the effect of polarity of stationary and mobile phases on retention of cannabinoids in normal phase liquid chromatography

## Introduction

In the last years, there has been an increased interest around the potential of cannabis-based products for medical and nutraceutical purposes. *Cannabis sativa* L., in particular, contains a large number of bioactive compounds, including flavonoids, terpenoids and, most importantly, cannabinoids, among which cannabidiol (CBD) and tetrahydrocannabinol (Δ^9^-THC) are the most popular and investigated ones. These two cannabinoids are not directly synthesized by the plant but they are produced after exposure to heat and light of their acid precursors (cannabidiolic acid, CBDA, and tetrahydrocannabinolic acid, THCA, respectively) [1–3], which represent the most abundant compounds naturally occurring in *Cannabis sativa* L..

Δ^9^-THC is known for its psychotropic effect. Its assumption, therefore, underlies strict regulations in many Countries. On the other hand, CBD does not get people high and it is not responsible for intoxicating effects. For this reason, it is one of the most studied and promising bioactive cannabinoids. Ongoing research is focused on the potential of CBD for the treatment of cancer, pain and many neurological diseases [4]. In addition, it possesses anti-inflammatory, anti-oxidant and anti-epilectics agents [5–8]. For the reasons above, the demand of pure CBD is continuously increasing and cannabis industry is demanding for efficient methods to separate and purify CBD from other components. However, purification of CBD from cannabis extracts could be challenging due to the complexity of the matrix, which includes other chemically similar cannabinoids, in addition to terpenes, waxes, etc. [9].

Preparative liquid chromatography is by far the most widely applied method in industry for the purification of single components from complex mixtures. The most important advantage of this technique is the great versatility that can be modulated through the combination of different adsorbents and eluents to achieve the separation of a wide range of compounds [10–13]. Several studies have already demonstrated that reversed-phase liquid chromatography (RPLC) can be efficiently applied for the separation and simultaneous quantification of a large number of cannabinoids [3, 14–20], at the point that both Dutch and German Pharmacopoeias report HPLC-UV as the official method for potency testing [21, 22]. Conversely, no fundamental studies about the employment of normal phase liquid chromatography (NPLC) for the separation of cannabinoids have been published so far with the exception of some works investigating the potential of NPLC for the chiral separation of cannabinoids on chiral stationary phases and some technical notes by Companies [19, 23–29]. On the opposite, being based on intrinsically different retention mechanisms compared to RPLC, NPLC might provide higher selectivity and resolution in some cases [30]. For instance when poor resolution of analytes under RP conditions is observed (e.g., the separation of the critical pair CBD-CBG [19]) or when impurities are more hydrophobic than the target analyte (in these cases, they are very strongly retained in RPLC, while could be quickly eluted in NPLC [31]) the employment of NPLC could be advantageous. Moreover, the use of apolar solvents facilitates sample preparation, especially of real samples. Indeed, in hexane or heptane the annoying issue of precipitation of apolar compounds (such as terpenes, abundandtly present in real samples of cannabis) is avoided. At the same time, sample solubility is increased in apolar solvents and therefore also column loading, while solvent removal from purified fractions is easier, which are both very important aspects from both a preparative and environmental viewpoint. Concerning sustainability of organic solvents, heptane and acetonitrile (which is commonly used in RPLC) both belong to the same class of “problematic” solvents [32], therefore the environmental impact of the two methods is almost the same. Finally, the use of low-viscosity solvents is less demanding in terms of pump back-pressure allowing for higher flow rates (i.e., faster runs).

Retention in NPLC has been usually described by the displacement model of retention for liquid-solid chromatography [33]. Briefly, the surface of the stationary phase is covered by a monolayer of solvent molecules that have to be displaced by the analyte molecule in order to be retained. In other words, solute and solvent molecules compete for adsorption on a limited number of adsorption sites. The understanding (and the prediction) of stationary phase selectivity in NPLC is a very complicated topic [30, 33–42]. The type of functional groups present on the stationary phase but also the nature of mobile phase modifier have a great effect on selectivity. The importance of hydrogen bonding has been recognized as one of pivotal aspects to be considered to understand retention and selectivity in NPLC [33, 34, 43–45].

Bare silica (Si) bears unbonded silanol groups (Si-OH) on the surface of the particles that are strong proton donors. They can interact via hydrogen bonding-type interactions with hydrogen bond acceptor groups (i.e., molecules with available electrons or a dipole moment). Cyano (CN), Amino (NH_2_) and Diol phases are monomeric phases directly attached to the silica surface through a flexible propyl linker. The linker is employed to favor the interactions between stationary phase and analytes and imparts a moderate hydrophobic character to the stationary phase. Moreover, on the polar-bonded phases, the presence of polar substituents on analytes is less important compared to bare silica, since unbonded silanols have been partially removed from the silica surface [31]. The CN stationary phase has a strong dipole that can interact with other dipoles present on solutes. Conversely, the NH_2_-based phase has a basic character, showing preferential retention for acidic solutes. The opposite happens on Diol phases [30, 46]. In this study, retention and selectivity of the four NP stationary phases Si, CN, NH_2_ and Diol have been investigated towards the separation of five neutral cannabinoids under normal phase conditions by varying the mobile phase modifier. Scope of the work is to understand retention mechanism of these compounds on polar stationary phases from a fundamental viewpoint. To the best of our knowledge, this is the first work reporting about a thorough investigation of retention mechanism of cannabinoids in NPLC. The columns have been also employed for the separation of a real cannabis sample.

## Experimental section

### Chemicals and solvents

Standard solutions (1 mg/mL) of cannabinoids were purchased from Cerilliant (Round Rock, TX, USA). Orthophosphoric acid, HPLC-grade solvents, including isopropanol (IPA), ethanol (EtOH), acetonitrile (ACN), heptane (Hept) and hexane (Hex) were from SigmaAldrich (St. Louis, MI, USA).

### Sample preparation

The plant material (female inflorescences) from a non-psychoactive *Cannabis sativa* variety (Gorilla Glue, indicated as G) was firstly submitted to a decarboxylation procedure. The inflorescences were kept at 110 ^∘^C for 15 min to remove volatile compounds; then, the temperature was raised to 120 ^∘^C for 60 min in order to totally turn cannabinoic acids into their neutral counterparts. Following a previous fully extraction optimized method [15], a portion of 0.25 g of plant material, previously deprived of seeds and twigs and properly grinded, were weighed and added with 10 mL of EtOH. The extraction of the target compounds was obtained by means of dynamic maceration at room temperature for 15 min. The extract was then paper-filtered and the residue was extracted twice more following the same procedure by adding 10 and 5 mL of EtOH, respectively. The filtrates of the three extractions were then combined and brought to the final volume of 25 mL with the extraction solvent.

### RPLC conditions

RPLC separations have been performed under reversed-phase conditions on a AZURA^*®*;^ HPLC system (KNAUER, Berlin, Germany) equipped with a binary high-pressure gradient pump (max pressure: 862 bars), a column thermostat, an autosampler and a photodiode array detector. A 150×4.6 mm Eurospher II 100-3 C18P column packed with 3 μm fully porous particles was used. Mobile phases were a phosphate buffer solution at pH = 2.2 and pure acetonitrile. The pump program is described in [47]. The wavelength was set at 228 nm. Injection volume was 2 μL. Calibration was performed using cannabinoid standards with known concentrations, ranging from 0.5 to 100 μg/mL.

### NPLC conditions

NPLC separations have been performed on a stainless steel AZURA^*®*;^ HPLC system (KNAUER, Berlin, Germany) for column screening equipped with a quaternary low pressure gradient pump (max pressure: 862 bars), a column thermostat, an autosampler, a photodiode array detector and two high-pressure multi-position valves (8 port) for column switching which provide automatted column screening. Four mobile phase compositions 95:5% v/v were used: (i) Hept/IPA, (ii) Hept/EtOH, (iii) Hex/IPA and (iv) Hex/EtOH. Four 150×4.0 mm Eurospher II 100-5 columns packed with 5 μm fully porous particles (100 Å pore size) have been tested: (i) NH_2_, (ii) Diol, (iii) CN and iv) Si (bare silica). The hold-up time of the columns was measured with toluene. Detection wavelength was 228 nm and temperature was set at 25 ^∘^C. Injection volumes for the sample and the standards were 5 μL and 3 μL, respectively. The flow rate was 1 mL/min.

## Results and discussion

Initially, the CBD-rich sample, extracted from non-psychoactive cannabis (G), was characterized by means of RPLC [47, 48], for the identification and quantification of the main cannabinoids. This information will be used later on to compare RP and NP chromatography.

**Fig. 1:**
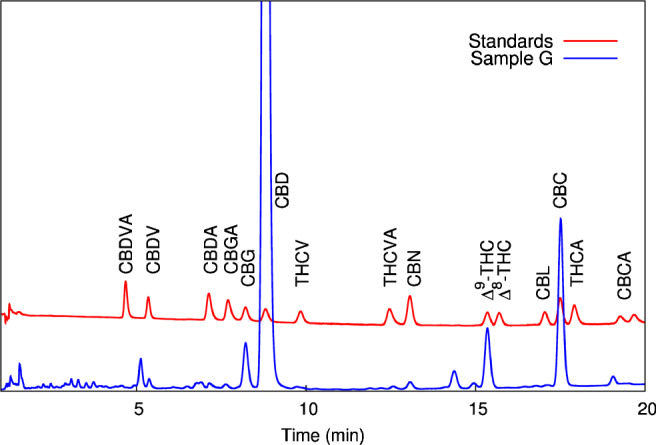
Analytical identification and quantification of the decarboxylated extract G obtained with reversed-phase chromatographic conditions (see Ref. [47] for details)

Through the comparison of retention times of cannabinoid standards, the identification of the following cannabinoids (in order of elution) was achieved: cannabidivarin (CBDV), cannabigerol (CBG), cannabidiol (CBD), cannabinol (CBN), tetrahydrocannabinol (Δ^9^-THC) and cannabichromene (CBC). Chromatograms of sample and standards are shown in Fig. 1. By means of calibration curves, the following weight percentages (% w/w) were obtained: CBDV: < 0.03%, CBG: 0.39%, CBD: 12.98%, THC: 0.52%, CBC: 0.67%, CBN: 0.03%.

**Fig. 2:**
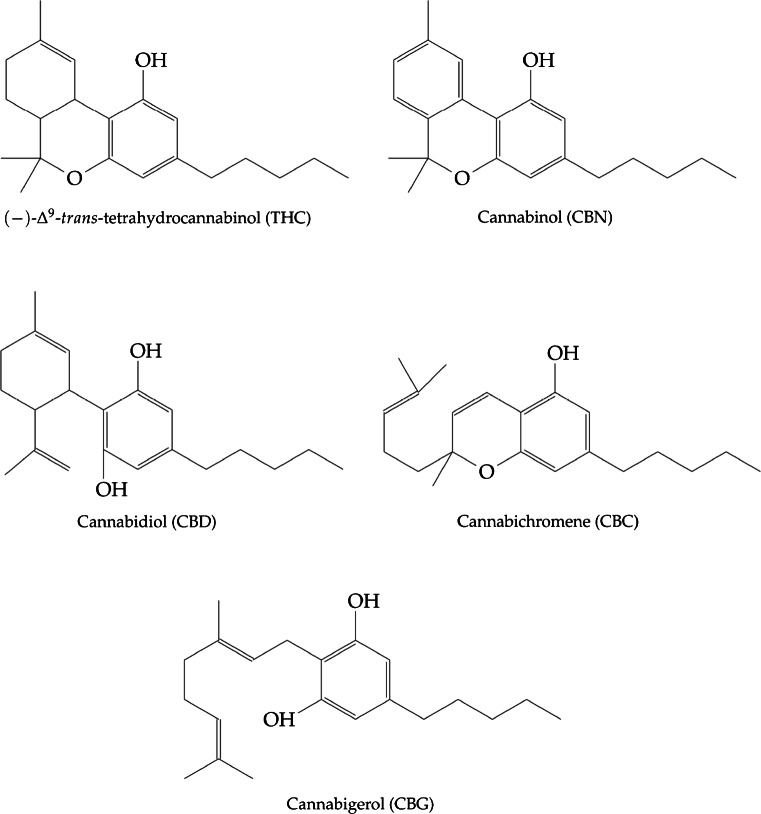
Chemical structure of the five main cannabinoids considered in this work

For the sake of simplification, only CBD, CBN, CBC, THC and CBG, i.e., cannabinoids with a content higher than 0.03% (w/w) have been further considered in this study. The chemical structures of these compounds are reported in Fig. 2.

### Influence of stationary phase on retention

**Fig. 3:**
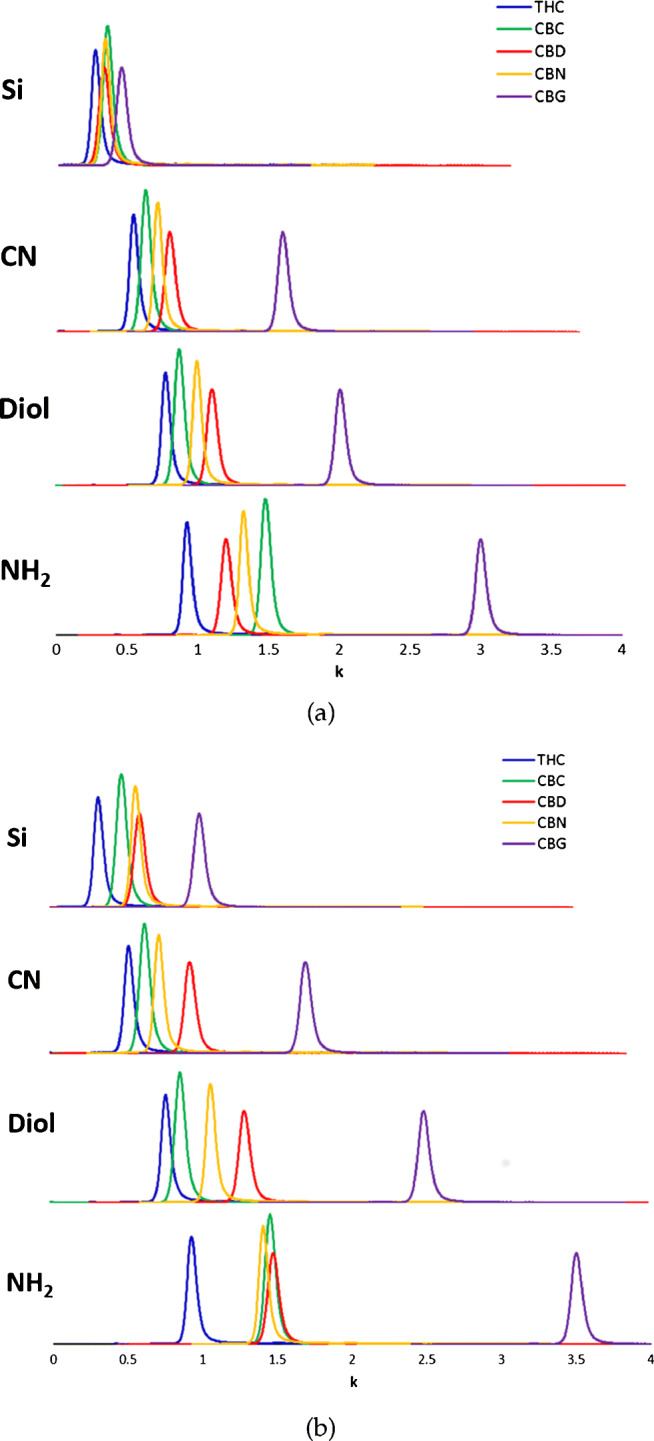
Chromatograms obtained with standard mixture of five cannabinoids (THC, CBC, CBD, CBN, CBG, 10 μg/mL) with 95:5% v/v heptane/isopropanol (**a**) and heptane/ethanol (**b**) on the four columns

The first part of this study focuses on the effect of the stationary phase on retention, at constant the mobile phase composition. Thus, Fig. 3 reports chromatograms of the five standards obtained on the four columns by employing Hept/IPA 95:5% v/v (Fig. 3a) and Hept/EtOH 95:5% v/v (Fig. 3b) as eluents. Table 1 summarizes the retention factors of the five cannabinoids under the different conditions.

**Table 1:** Retention factors (*k*) measured on the four columns with the different MP compositions through the injection of standard solutions of the five main cannabinoids found in the cannabis extract

MP (95:5%)	Column	*k*_*CBD*_	*k*_*CBN*_	*k*_*CBC*_	*k*_*CBG*_	$k_{{\Delta }^{9}-THC}$
Hept/IPA	NH_2_	1.16	1.32	1.54	3.01	0.92
	CN	0.80	0.70	0.61	1.61	0.54
	Diol	1.08	1.05	0.90	2.08	0.79
	Si	0.32	0.33	0.34	0.46	0.26
Hept/EtOH	NH_2_	1.45	1.39	1.44	3.54	0.95
	CN	0.89	0.71	0.58	1.73	0.55
	Diol	1.30	1.11	0.90	2.54	0.83
	Si	0.61	0.56	0.51	1.01	0.44
Hex/IPA	NH_2_	1.12	1.32	1.54	2.80	0.92
	CN	0.80	0.71	0.62	1.54	0.57
	Diol	1.05	1.02	0.90	1.95	0.80
	Si	0.33	0.34	0.35	0.45	0.29
Hex/EtOH	NH_2_	1.42	1.43	1.48	3.26	0.99
	CN	0.85	0.70	0.58	1.61	0.56
	Diol	1.23	1.12	0.88	2.32	0.81
	Si	0.55	0.55	0.48	0.88	0.42

By comparing what happened in RPLC, the first interesting observation is that in NPLC a quasi-complete reversal of elution order of cannabinoids was obtained. Indeed, as shown in Fig. 3, in NPLC on all columns but on the NH_2_ one, THC is the first eluted cannabinoid followed by CBC, CBN, CBD and CBG. On the NH_2_ column, cannabinoids are eluted as follows: THC, CBD, CBN, CBC and CBG (see Fig. 3a). In all cases, the NH_2_ column was the most retentive for all cannabinoids, followed by Diol, CN and Si columns. This retention behavior can be tentatively explained by considering the interplay of different factors including the polarity of the electron-rich functional groups present on the surface of stationary phases, the strength of the mobile phase and the characteristics of cannabinoids, in particular in relationship to their ability to behave as H bond acceptors-donors (Fig. 2). Indeed, in NPLC retention and selectivity depend in a complex way on the stationary phase type (i.e., functional groups present on the surface of adsorbents) and solvent composition (i.e., amount and type of strong polar modifier) [31, 35, 44, 49–52]. Solute and mobile phase molecules interact with polar adsorption sites present on the surface of adsorbents by competitive, polar and hydrogen bonding-type interactions. In this study, mobile phase is a mixture of an apolar solvent and an alcohol, which is a very strong, proton-donor solvent. On the other hand, analytes are weaker proton donors and less polar molecules (see Fig. 2). It is therefore reasonable to assume that a monolayer of alcohol molecules is adsorbed on the surface of the stationary phase via strong hydrogen bonding-type interactions. This leads to the deactivation of silanols, with important consequences on retention [30, 31, 53]. This aspect is particularly relevant on the bare silica column, where indeed retention of cannabinoids is very weak (Fig. 3). Conversely, even if residual silanols are deactivated by stronger solvents [30, 54, 55] also on the bonded-phase columns, the retention on these is stronger thanks to the presence of polar and apolar interactions between analytes and functional groups on the stationary phase. The CN column shows slightly larger retention compared to silica one, thanks to *π*-*π* and hydrophobic interactions between analytes and functional groups. On the other hand, retention on CN column is weaker compared to both NH_2_ and Diol columns, since the CN group lacks hydrogen bond donor capability. CN group can establish strong dipole-dipole interactions with dipolar solutes (e.g., nitriles and nitro compounds) [30, 56, 57], which is not the case of cannabinoids. Both Diol and NH_2_ columns are hybrid stationary phases (indeed they can be used both as RPLC and NPLC adsorbents, depending on the polarity of the mobile phase). Therein retention mechanisms are mainly based on both hydrophobic and hydrophilic interactions. NH_2_ column, being a basic adsorbent with available electrons, preferentially interacts with proton-donor acidic groups [31, 35, 40, 58], such as hydroxy ones. This can explain the stronger retention of cannabinoids observed on the NH_2_ column. Diol column exhibits an intermediate behavior between CN and NH_2_ groups, as reported in Ref. [30].

In summary, the overall retention on the four stationary phases is the following: NH_2_ > Diol > CN > Si.

Analytes with different spatial configuration, steric hindrance and number of electronegative atoms will interact with these adsorbents to different extents. As a consequence, their ability to form hydrogen bonds or to establish hydrophobic interactions with the functional groups of the stationary phase will have a direct influence on retention and selectivity. A simple comparison between the chemical structures of the five cannabinoids under study, reported in Fig. 2, may give some insights about differences in interaction and adsorption mechanisms with the stationary phases. Retention factors of THC, CBN, CBC and CBD are very close for all the experimental conditions (Table 1), indeed they have very similar chemical structures containing at least two cyclic structures. THC and CBN show slightly different retention behavior (see Fig. 3 and Table 1), even if their structures only differ in the number of *π*-bonds. The larger availability of *π*-electrons imparts more polar character to CBN compared to THC, leading to a relatively larger retention. The more open and flexible structure of CBG allows for higher degree of interaction between the two hydroxy groups and the functional groups present on bonded stationary phases, leading to larger retention with respect to all other cannabinoids. CBD shows weaker retention relative to CBG, even if it bears the same number of free hydroxy groups. One possible explanation can be the steric hindrance given by the disubstituted cyclohexene linked to the diphenol in the structure of CBD.

### Effect of mobile phase composition on retention and selectivity

**Fig. 4:**
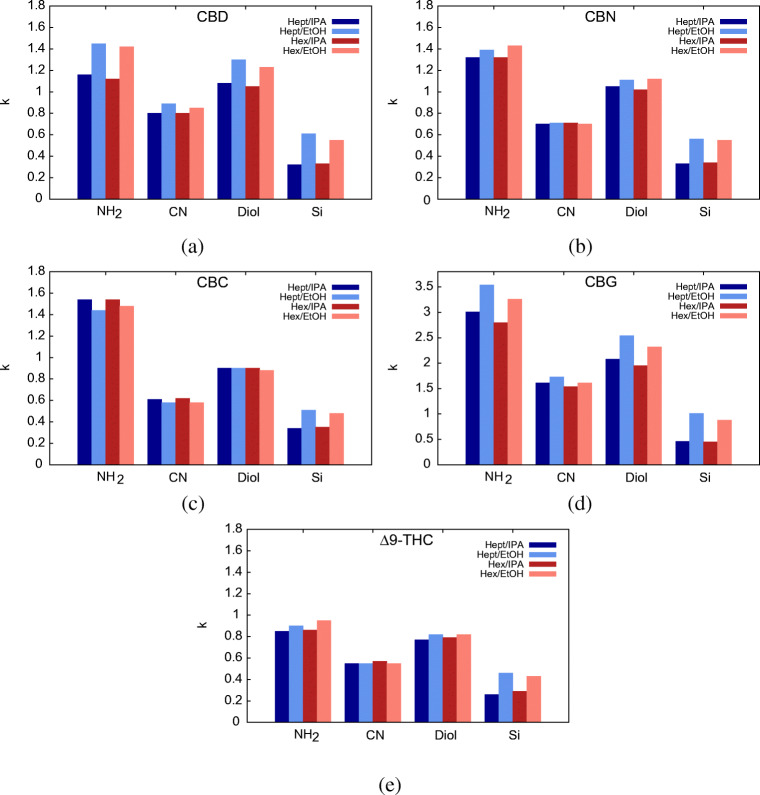
Bar plots of retention factors measured for the five main cannabinoids (**a** CBD, **b** CBN, **c** CBC, **d** CBG and **e** THC) on NH_2_, CN, Diol and Si columns with four different mobile phase compositions (95:5% v/v). Light color bars: ethanol as polar modifier; dark color bars: isopropanol as polar modifier; red bars: hexane as apolar solvent; blue bars: heptane as apolar solvent

The second part of the study regards the investigation of changes in retention and selectivity by varying both the nonpolar component of the mobile phase (Hex or Hept) and the alcohol (EtOH or IPA). The percentage ratio of the two solvent was 95:5% (v/v) nonpolar eluent/alcohol in each case. Retention factors of the five cannabinoids on the four columns with the four different eluent combinations are graphically represented in Fig 4. From these plots, it can be evinced that, independently on the stationary phase, the presence of EtOH (in place of IPA) increases retention for almost all cannabinoids. Only exceptions are CBN and THC on the CN column, for which retention does not practically change by changing the alcohol, CBC on the Diol column (again, unchanged retention no matter the alcohol type) and CBC on NH_2_ column, where on the opposite a larger retention was observed with IPA. CBD and CBG seem to be most sensitive to changes in the polar modifier of the mobile phase, probably due to the presence of two hydroxy groups in their structure, while CBN, CBC and Δ^9^-THC are less affected. Another interesting feature that can be evinced from these plots is that the apolar solvent (hexane or heptane), has a negliglible influence on the retention of all the cannabinoids.

**Fig. 5:**
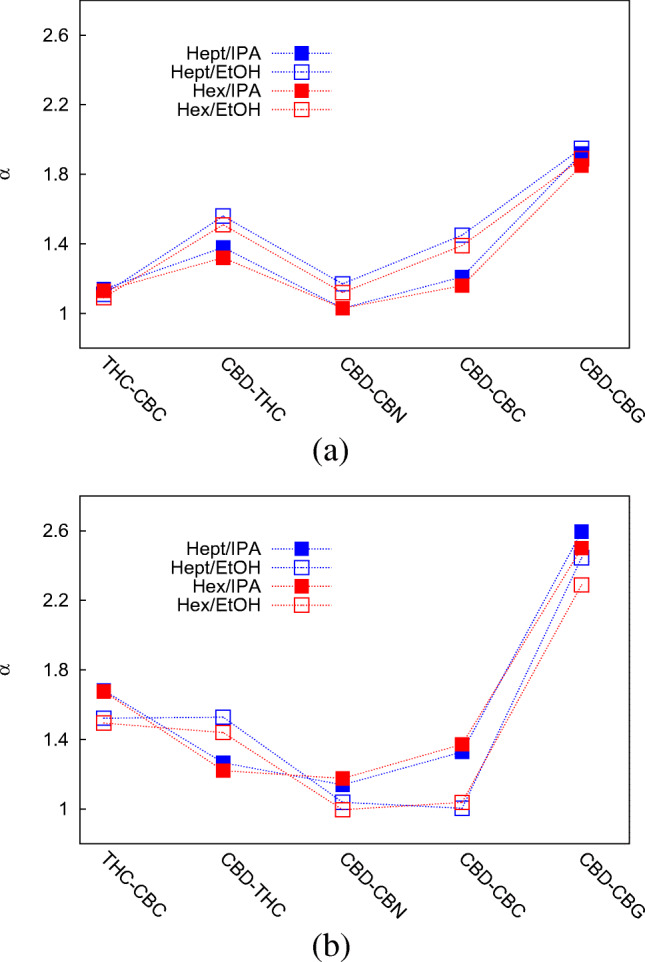
Selectivity values measured for 5 couples of cannabinoids on Diol (**a**) and NH_2_ (**b**) columns for the four MP compositions under study (see legend for details)

The dependence of the selectivity (*α* = *k*_2_/*k*_1_, with *k*_2_ the retention factor of the more retained compound and *k*_1_ that of the less retained one) on the MP composition has also been investigated. Data are listed in Table 2 and graphically reported in Fig. 5 for Diol and NH_2_ columns as an example (data related to CN column follow almost the same trend of Diol column and data related to Si column are not significant, being the separation not optimal). From these data, a clear distinction is observed between selectivity values obtained with EtOH and IPA, with no influence of the apolar solvent, as expected.

**Table 2:** Selectivity values (*α*) measured on the four columns at the different MP compositions

MP (95:5%)	Column	*α* THC-CBC	*α* CBD-THC	*α* CBD-CBN	*α* CBD-CBC	*α* CBD-CBG
Hept/IPA	NH_2_	1.68	1.27	1.14	1.33	2.59
	CN	1.12	1.48	1.15	1.32	2.01
	Diol	1.14	1.38	1.03	1.21	1.92
	Si	1.33	1.23	1.04	1.08	1.45
Hept/EtOH	NH_2_	1.52	1.53	1.04	1.00	2.44
	CN	1.05	1.61	1.26	1.53	1.94
	Diol	1.11	1.56	1.17	1.45	1.95
	Si	1.52	1.84	1.10	1.21	1.64
Hex/IPA	NH_2_	1.68	1.22	1.18	1.37	2.50
	CN	1.08	1.40	1.13	1.29	1.93
	Diol	1.13	1.32	1.03	1.16	1.85
	Si	1.23	1.17	1.02	1.05	1.34
Hex/EtOH	NH_2_	1.49	1.44	1.00	1.04	2.29
	CN	1.03	1.51	1.21	1.46	1.90
	Diol	1.09	1.51	1.12	1.39	1.89
	Si	1.16	1.33	1.00	1.15	1.58

Five pairs of cannabinoids have been selected: THC-CBC, CBD-THC, CBD-CBN, CBD-CBC and CBD-CBG

These aspects can be explained by taking into account differences in the polarity scales (or indexes) of solvents. These scales list solvents in order of polarity, by considering the sum of all molecular properties responsible for all the interaction forces between solvent and solute molecules, ranging from apolar (tetramethylsilane with index = 0.000) to polar solvents (water with index = 1.000) [59]. In this specific case, hexane and heptane show very close values (0.009 and 0.012), while polarity indexes of ethanol and isopropanol significantly differ (0.654 and 0.546), leading to more marked changes in retention and selectivity. A possible explanation of the smaller retention observed when IPA is employed as the polar modifier could be that IPA, even though less polar than EtOH, is however able to interact more strongly with polar functional groups linked through apolar propyl bridges on bonded stationary phases. Thus, to be retained, analytes must displace one or more strongly adsorbed IPA molecules from the stationary phase. On the contrary, the displacement of EtOH molecules, less strongly adsorbed, would be easier for the solute, leading to larger retention in most cases.

From Fig. 5, it can be evinced that both Diol and NH_2_ columns show opposite trends for all the couples of cannabinoids but CBD-THC, for which mobile phases containing EtOH lead to larger selectivity compared to IPA. The lack of selectivity observed for the couples CBD-CBN and CBD-CBC on the NH_2_ column with EtOH employed as strong MP modifier is probably due to the higher dependence of CBD retention on the nature of the polar modifier, possibly due to a different solvatation in the MP. Indeed, CBD retention shows a 20% change when passing from one alcohol to the other, while for CBN and CBC the change is only roughly 5%.

### Application to real samples

**Fig. 6:**
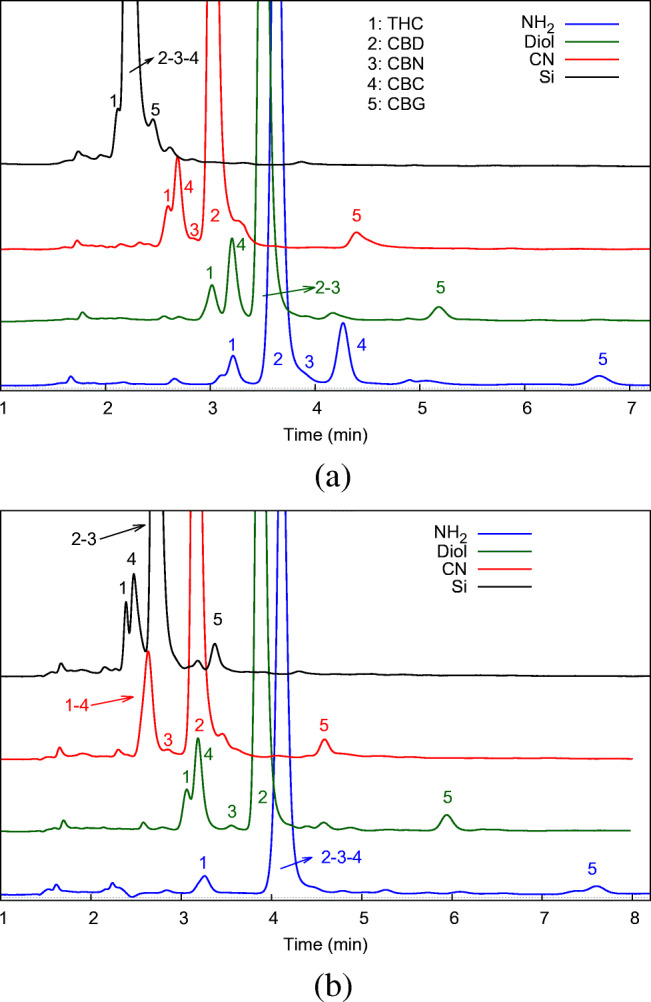
Chromatograms of sample G obtained with 95:5% v/v Hept/IPA (**a**) and Hept/EtOH (**b**) on NH_2_ (blue), CN (red), Diol (green) and Si (black) columns. 1: Δ^9^-THC, 2: CBD, 3: CBN, 4: CBC and 5: CBG

The same experimental conditions have been applied to the separation of a real sample (“Sample preparation”) in order to have some insights about the possible effects on the separation coming from the presence of other compounds in the real matrix, on the one hand, and the relative abundance of cannabinoids, on the other. Figure 6 reports cromatograms measured on the entire set of columns with Hept/IPA and Hept/EtOH as mobile phases for the cannabis extract G. This sample has been previously fully characterized using a fully validated analytical method [48]. In most cases the five cannabinoids are well resolved and can be easily identified. On the Diol column operated with Hept/IPA as mobile phase, the tiny CBN peak can not be distinguished from the more concentrated CBD peak. However, this issue can be easily overcome by changing the polar modifier in the mobile phase (see Fig. 6b). Another interesting observation concerns the NH_2_ column. The use of IPA as polar modifier allows not only to identify all five cannabinoids, but also to partially resolve unknown species eluting before the THC peak (number 1 in the chromatogram), which cannot be observed with EtOH.

## Conclusions

Efficient methods for the separation and the purification of cannabinoids from different cannabis extracts are among the primary needs of industry. Even if RPLC represents the most widely applied chromatographic mode to separate this class of compounds, novel approaches and conditions need to be explored to facilitate sample preparation and to shorten run times. NPLC could offer several advantages in this sense, including better conditions for solvent evaporation and sample concentration in preparative conditions.

This work has been intended to start the investigation about the possibility of separating the main neutral cannabinoids by means of NPLC. The polar adsorbents employed have been demonstrated to be promising candidates, with the only exception of the silica column. By optimizing mobile phase conditions (not a scope of this work), it can be possible to achieve good resolution of the main cannabinoids. The greatest advantage would be shorter analysis times and the possibility to use higher flow rates thanks to a low-viscosity mobile phase. In particular, the NH_2_ adsorbent already showed an acceptable separation of the five main cannabinoids by using Hept/IPA 95:5% (v/v) as mobile phase.

Results of this work provide important information not only for the development of analytical methods for separation of cannabinoids in NPLC, but also for the setting up of purification procedures, including continuous approaches such as Simulated Moving Bed (SMB). This technique has been already applied for the purification of CBD (up to 87% purity) [60, 61] and Δ^9^-THC (99% purity) [62] from complex matrices. SMB works in isocratic conditions and it would benefit of short run times and low-viscosity solvents, as those employed for the screening presented in this work.
